# *Lactobacillus paragasseri* ameliorates age-dependent thermotaxis decline in *Caenorhabditis elegans*

**DOI:** 10.1038/s41598-026-48002-7

**Published:** 2026-04-17

**Authors:** Masaru Tanaka, Sachio Tsukada, Moon Sun Jang, Binta Maria Aleogho, Ikue Mori, Kentaro Noma

**Affiliations:** 1 Milk Science Research Institute, MEGMILK SNOW BRAND Co. Ltd.,, 1-1-2 Minamidai, 350-1165 Kawagoe, Saitama, Japan; 2 Group of Nutritional Neuroscience, Neuroscience Institute, Graduate School of Science, Nagoya University, Nagoya, 464-8602 Japan; 3 Group of Molecular Neurobiology, Neuroscience Institute, Graduate School of Science, Nagoya University, 464-8602 Nagoya, Japan; 4 Group of Microbial Motility, Department of Biological Science, Division of Natural Science, Graduate school of Science, Nagoya University, 464-8602 Nagoya, Japan; 5 Chinese Institute for Brain Research, Changping District, 102206 Beijing, China; 6 Graduate School of Science, Nagoya City University, Mizuho-ku, 467-8501 Nagoya, Japan

**Keywords:** Biological techniques, Microbiology, Neuroscience

## Abstract

Diet can modulate cognitive aging, but the underlying mechanisms are poorly understood. Here, we show that specific lactic acid bacteria (LAB) ameliorate age-dependent decline of associative learning behavior in the nematode *Caenorhabditis elegans*. Screening of LAB and bifidobacterial species, mixed with the standard *Escherichia coli* (*E. coli*) diet, revealed that *Lactobacillus paragasseri* (*Lb. paragasseri*) protects against behavioral aging without affecting growth, lipid storage, or lifespan. Different treatment of bacteria suggested that *Lb. paragasseri* contains heat-labile, yet freeze-stable bacterial components responsible for the beneficial effect. Calcium imaging revealed that *Lb. paragasseri* suppresses age-associated hyperactivation of AWC sensory neurons, a key driver of behavioral decline. Furthermore, the influence of *Lb. paragasseri* required the FOXO transcription factor DAF-16. Proteomic profiling identified differentially expressed proteins between aged animals fed *E. coli* alone and those fed a mixture of *E. coli* and *Lb. paragasseri*. Together, these findings highlight *Lb. paragasseri* as a potential microbiota-based intervention for preserving neural function during aging.

**Supplementary Information:**

The online version contains supplementary material available at 10.1038/s41598-026-48002-7.

## Introduction

The decline in both physical and cognitive abilities is an inevitable aspect of aging across most animals, and concerns about cognitive decline have grown in our society with increasing longevity^1^. Aging causes changes in the nervous system, including neuronal aging and cognitive deterioration. Aging neurons are thought to experience a disruption in cellular homeostasis due to the accumulation of amyloid-beta and phosphorylated tau, along with ongoing oxidative stress and inflammation^2^. The continuation of these conditions may result in cognitive impairment and dementia^2^. Current treatments for dementia, such as acetylcholinesterase inhibitors and NMDA receptor antagonists, aim to slow disease progression^3^, but rarely achieve full recovery. Moreover, long-term pharmacotherapy presents significant burdens—physical, financial, and emotional—on patients and caregivers. These limitations underscore the need for more economical, convenient, and sustainable preventative measures against age-dependent cognitive decline.

Non-pharmacological interventions, including regular exercise, dietary modifications, and cognitive training, have shown promise in mitigating age-related cognitive decline^4,5^. Among these, dietary interventions using lactic acid bacteria (LAB) and bifidobacteria are particularly attractive. These lactic acid-producing Gram-positive bacteria, commonly found in fermented dairy products, have long been recognized for their health benefits, such as cognitive enhancement^6,7^. For example, clinical studies highlight the roles of *Bifidobacterium breve* and *Lactobacillus rhamnosus* GG, pivotal components of gut microbiota, in supporting cognitive function^8,9^. Unlike pharmacological treatments, dietary interventions using commensal bacteria act more gradually and can be initiated even before the onset of pathological symptoms. They therefore represent more sustainable and economical solutions for cognitive decline in aging populations. However, the precise mechanisms by which bacteria influence age-dependent cognitive decline remain poorly understood.

*C. elegans* is a powerful model for dissecting the cellular and molecular mechanisms of aging due to its short lifespan, genetic tractability, and wide range of available genetic tools. It is also well-suited for studying cognitive functions, as it exhibits complex associative learning behaviors despite having a compact nervous system with only 302 neurons^10^. Because *C. elegans* consumes bacteria as food, dietary interventions using different bacteria can be studied in relation to physiology and behavior. In laboratory settings, the *E. coli* strain OP50 is the standard diet^11^, although *C. elegans* can feed on a variety of bacterial species, including lactic acid bacteria and bifidobacteria^12^. These features make *C. elegans* an excellent system for investigating how diet influences age-dependent cognitive decline. In particular, we focused on associative learning between temperature and food. *C. elegans* can be cultivated at a non-noxious temperature within the range of 15–25 °C. When animals are cultivated with food and placed on a thermal gradient without food, they migrate toward their prior cultivation temperature. This behavior is called thermotaxis^13,14^. By contrast, when cultivated without food, they fail to migrate toward the previous cultivation temperature^14^. The underlying cellular and molecular mechanisms of thermotaxis are well characterized in young animals^15–19^. Temperature is sensed and memorized by AFD sensory neurons, which drive thermophilic and cryophilic behaviors through AIY and AIZ interneurons, respectively^13,20^. The animals’ satiety state is incorporated into this circuit through the insulin/IGF-1 neuropeptide, INS-1, which is received by the thermosensory neuron AWC^21–23^. Consequently, *ins-1* mutants are defective in avoidance behavior from the cultivation temperature under starvation conditions^22,23^.

When *C. elegans* is cultivated on the standard laboratory diet of *E. coli*, they exhibit age-dependent thermotaxis decline by Day 5 of adulthood^24,25^. However, AFD and AIY activities are not significantly decreased in aged animals^26^. Instead, the thermotaxis decline is attributed to the age-dependent spontaneous hyperactivation of AWC sensory neurons and AIA interneurons, independent of a temperature stimulus^26^. Previously, we screened 35 lactic bacteria and bifidobacteria to identify strains that could prevent this decline^25^. In the study, animals were cultivated with *E. coli* during development until Day 1 of adulthood (Day 1), and then the diet was switched to a single lactic acid bacterium without *E. coli* from Day 1 to Day 5. We found that *Limosilactobacillus reuteri* (*Lm. reuteri)* suppressed thermotaxis decline without affecting lifespan. Note that *Lm. reuteri* was previously referred to as *Lactobacillus reuteri*, but it was renamed *Limosilactobacillus reuteri*^27^. *Lm. reuteri* is a rod-shaped, heterofermentative bacterium that produces lactic acid as well as acetic acid, ethanol, and CO_2_ through fermentation. High thermotaxis ability of *Lm. reuteri*-fed aged animals depends on the FOXO transcription factor DAF-16 in *C. elegans*^25^. However, when mixed with *E. coli*, *Lm. reuteri* did not show any beneficial effect. Furthermore, *Lm. reuteri* did not support the growth of animals from eggs, and animals fed *Lm. reuteri* from Day 1 to Day 5 exhibited a slightly paler body. These observations raised the concern that its effect may involve indirect metabolic alterations rather than beneficial components.

In this study, we supported normal animal growth with the standard diet, *E. coli*, and sought to identify beneficial bacteria capable of preventing age-dependent thermotaxis decline, even when mixed with *E. coli* as an additive. We screened 51 lactic acid bacteria and bifidobacteria mixed with *E. coli* and found that *Lactobacillus paragasseri* (*Lb. paragasseri*) could ameliorate age-dependent thermotaxis decline. The *E. coli* + *Lb. paragasseri* mixture supported normal growth, and calcium imaging revealed that *Lb. paragasseri* suppressed age-dependent hyperactivation of AWC sensory neurons. Like the effects of *Lm. reuteri*, the beneficial effect of the *E. coli* + *Lb. paragasseri* mixture depended on *daf-16*. Furthermore, proteomic analysis identified differentially expressed proteins between *E. coli*-fed and *E. coli* + *Lb. paragasseri-*fed Day 5 animals. These findings highlight *Lb. paragasseri* as a beneficial bacterium that mitigates age-dependent thermotaxis decline and shed light on its underlying cellular and molecular mechanisms.

## Results

### *Lb. paragasseri* and *P. stilesii* prevent age-dependent thermotaxis decline even when mixed with *E. coli*

**Fig. 1:**
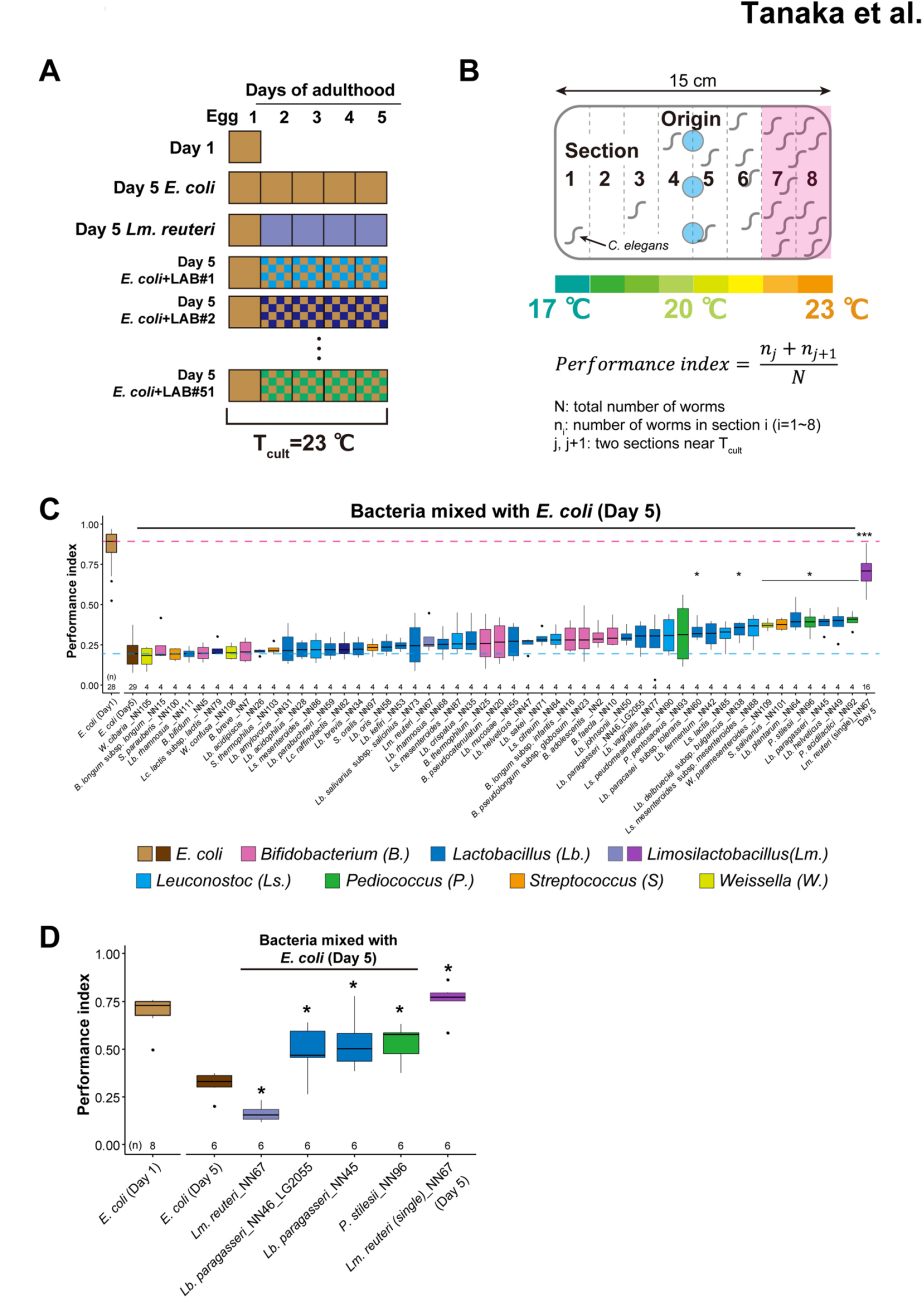
Bacterial screening based on the thermotaxis behavior of aged *C. elegans* **(A)** Schematic of bacterial feeding procedure. Animals were fed the standard laboratory diet, *E. coli*, from eggs to Day 1 of adulthood. Day 1 animals were transferred to *E. coli*, *Lm. reuteri*, or a 1:2 mixture of *E. coli* and candidate bacteria. Animals were transferred daily to new plates, cultivated until Day 5, and subjected to thermotaxis assays at Day 5. Cultivation temperature (T_cult_) was 23 °C. **(B)** Schematic of the thermotaxis assay. Animals were placed at the three starting points (light blue circles) along the center of an assay plate and allowed to migrate on a thermal gradient between 17 °C and 23 °C without food. After 1 h, the number of animals in each of the eight sections was counted, and the thermotaxis performance index was calculated using the indicated formula. The two sections near the T_cult_ are highlighted in pink. In this study, we calculated the index as the number of animals in Sects.  7 and 8 divided by the total number of animals. **(C)** Screening of LAB that affect thermotaxis in aged animals when mixed with *E. coli*. *E. coli*-fed Day 1, *E. coli*-fed Day 5, and *Lm. reuteri*-fed Day 5 animals serve as controls. Forty-two LAB or nine *Bifidobacterium* species were mixed with *E. coli* as described in (A), and the thermotaxis performance of Day 5 animals was measured. Sample size (n) indicates the number of assays. Light blue and light pink dotted lines indicate the median of performance indices of *E. coli*-fed Day 1 and *E. coli*-fed Day 5 animals, respectively. **(D)** Thermotaxis performance of Day 5 animals fed *E. coli* + *P. stilesii* or *E. coli* + *Lb. paragasseri*. One strain of *P. stilesii* and two strains of *Lb. paragasseri* were tested. Statistics: Mann–Whitney U test was used to compare each data with the *E. coli*-fed Day 5 condition, and the p-values were adjusted using the Benjamini-Hochberg method. *p < 0.05.

We evaluated associative learning ability by cultivating animals at 23 °C with food and subsequently examining their thermotaxis behavior on assay plates with a thermal gradient from 17 °C to 23 °C (Fig. 1A and B). Animals were placed at the center of the plate, where the temperature is 20 °C (Fig. 1B). As in our previous report^25^, young animals (Day 1 of adulthood) cultivated with the standard laboratory diet, *E. coli*, displayed robust thermotaxis behavior whereas aged animals (Day 5 of adulthood) exhibited thermotaxis defects (Fig. 1C, *E*. *coli*). To examine the influence of bacteria on the behavior of aged animals independently of developmental effects, animals were first grown on *E. coli* until Day 1, after which the diet was switched to another bacterium. Similar to our previous study^25^, switching the diet to *Lm. reuteri* alone prevented the age-dependent thermotaxis decline (Fig. 1A and C, *Lm. reuteri* (single)_NN67, rightmost box plot). However, when animals were fed a mixture of *E. coli* and *Lm. reuteri*, they still showed age-dependent decline (Fig. 1C, *Lm. reuteri*_NN67)^25^. Thus, we could not conclude that *Lm. reuteri* itself contained anti-aging factors in our previous study^25^. These observations motivated us to identify bacterial species capable of ameliorating the age-dependent thermotaxis decline even when mixed with *E. coli*.

Animals were cultivated on *E. coli* until Day 1 and then transferred to plates containing a mixture of *E. coli* and a test bacterial strain, focusing on LAB and bifidobacteria (Fig. 1A). We first screened 42 LAB—23 *Lactobacilli (Lb.)*, 1 *Limosilactobacillus (Lm.*), 3 *Pediococci (P.)*, 2 *Lactococci (Lc.)*, 4 *Streptococci (S.)*, 6 *Leuconostoc (Ls.)*, and 3 *Weissella (W.)—* and 9 *Bifidobacteria (B.)* species (Supplementary Table 1, Fig. 1A and C). To confirm that animals ingested these LAB even when mixed with *E. coli*, we observed animals fed mixtures of fluorescently labeled LAB and unlabeled *E. coli*. As a control, we fluorescently labeled *E. coli* and observed them in the intestine when mixed with unlabeled *E. coli* (Supplementary Fig. 1, *E*. *coli*). Despite variations in fluorescence intensity, intestinal fluorescence was consistently detected across all conditions, confirming that animals ingested the screened bacteria (Supplementary Fig. 1). We selected 12 bacteria from the 51 tested in the initial screen based on their higher thermotaxis at Day 5 (Fig. 1C). The first screen might have false positives and false negatives due to large variation and a small sample size. Thus, the 12 candidates were further narrowed down in the second (Supplementary Fig. 2A) and third screens (Supplementary Fig. 2B). Among these screens, *Lactobacillus paragasseri* (*Lb. paragasseri*, NN45) and *Pediococcus stilesii* (*P. stilesii*, NN96) consistently ameliorated thermotaxis decline in Day 5 animals (Fig. 1C and Supplementary Fig. 2), and we confirmed the effect in independent trials (Fig. 1D). Subsequently, we investigated whether the ameliorative effect was strain-dependent within the hit species. Although another strain of *Lb. paragasseri* (NN46) did not show a significant effect in the first screen, and was not included in the second and third screens, we additionally tested *Lb. paragasseri* (NN46) together with NN45 in the same experimental batch as an independent validation (Fig. 1D). Under these conditions, *Lb. paragasseri* (NN46) exerted an ameliorative effect comparable to *Lb. paragasseri* (NN45) (Fig. 1D). This result supports that the beneficial effect of *Lb. paragasseri* is not restricted to a single strain, and the absence of NN46’s effect in the first screen could be due to variation. On the other hand, we did not have other strains of *P. stilesii* available and thus could not assess strain specificity.

**Fig. 2:**
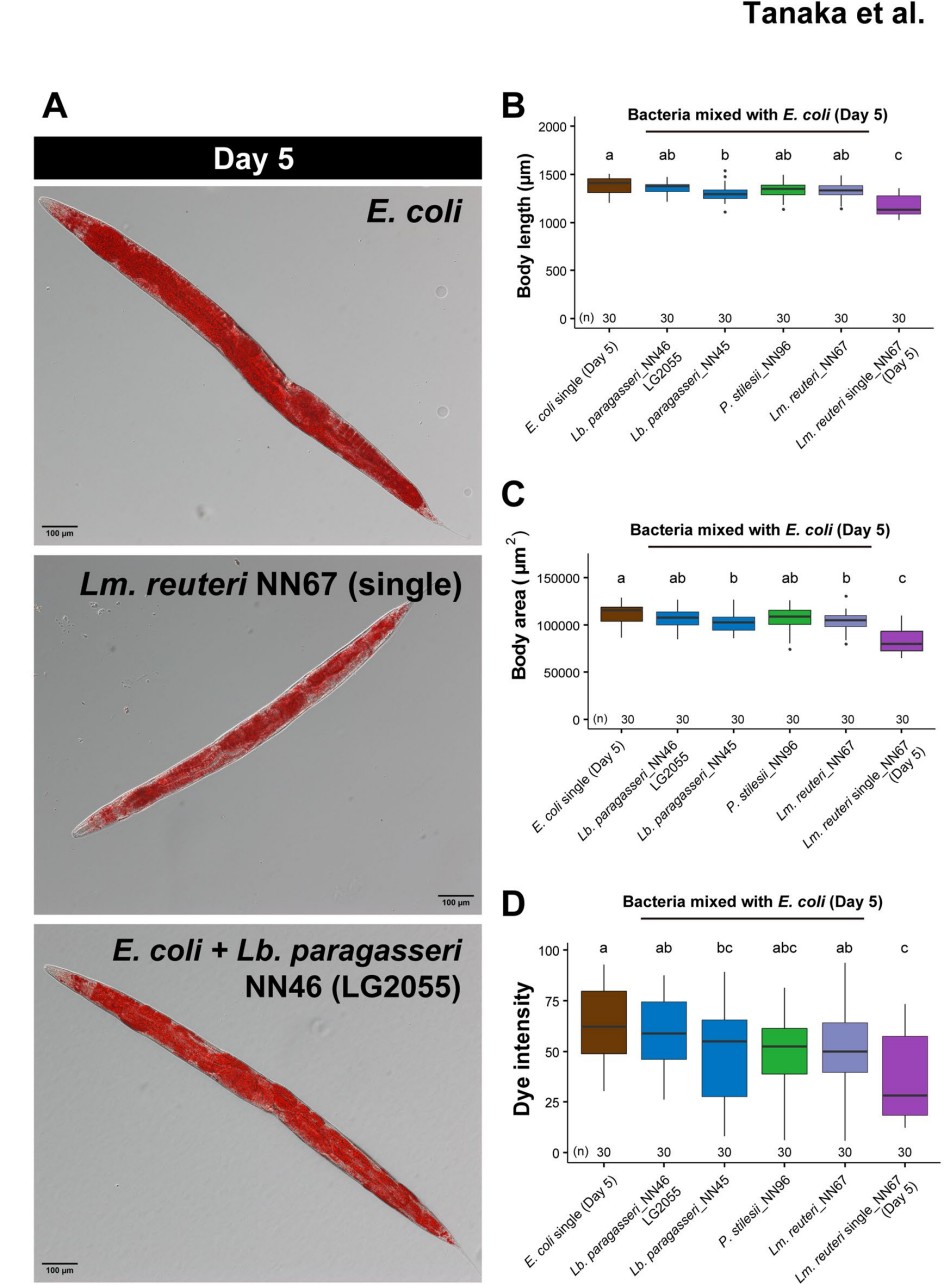
The mixture of *E. coli* and *Lb. paragasseri* (NN46) supports the normal physiology of *C. elegans* during aging Day 5 animals were fed the indicated bacteria from Day 1. **(A)** Representative bright-field images of animals stained with Oil Red O. Scale bar = 100 μm. **(B)** Body length of animals. **(C)** Body area of animals.**(D)** Quantified Oil Red O staining intensity. Statistics: Sample size (n) indicates the number of animals examined. Tukey multiple-comparison test was used to compare all possible pairs. Different letters indicate statistical difference (*p* < 0.05).

Next, we examined whether feeding with *Lb. paragasseri* (NN46) and *P. stilesii* (NN96) affected the animals’ physiology. To investigate whether the mixture of *E. coli* and LAB supports normal growth and lipid accumulation, we treated animals with Oil Red O (ORO) dye, which stains neutral lipids red^28^ and measured body length, body area, and intensity of the ORO staining in Day 5 animals fed *E. coli*, *Lm. reuteri*, and bacterial mixtures of *E. coli* and *Lb. paragasseri* or *P. stilesii.* Day 5 animals fed *Lm. reuteri* alone had shorter body length and smaller body area than those fed *E. coli* (Fig. 2A-C, *Lm. reuteri*, single NN67); lipid levels were also lower in *Lm. reuteri-*fed animals (Fig. 2A and D, *Lm. reuteri*, single NN67*)*. These quantifications were consistent with our previous observations^25^. By contrast, Day 5 animals fed mixtures of *E. coli* and LAB (*Lb. paragasseri*, *P. stilesii*, or *Lm. reuteri*) had relatively normal body length and area (Fig. 2A-C) and showed improved lipid accumulation, compared to those fed only *Lm. reuteri* (Fig. 2A and D). Among these LAB, animals fed *E. coli* + *Lb. paragasseri* (NN46) had body length, body area, and lipid accumulation comparable to those of animals fed the standard diet, *E. coli*. In summary, feeding animals with the mixture of *E. coli* and *Lb. paragasseri* (NN46) supports normal growth and lipid accumulation without causing any detectable physiological abnormalities, in contrast to monoxenic feeding with *Lm. reuteri*.

### *Lb. paragasseri* affects thermotaxis possibly through heat-labile metabolites

**Fig. 3:**
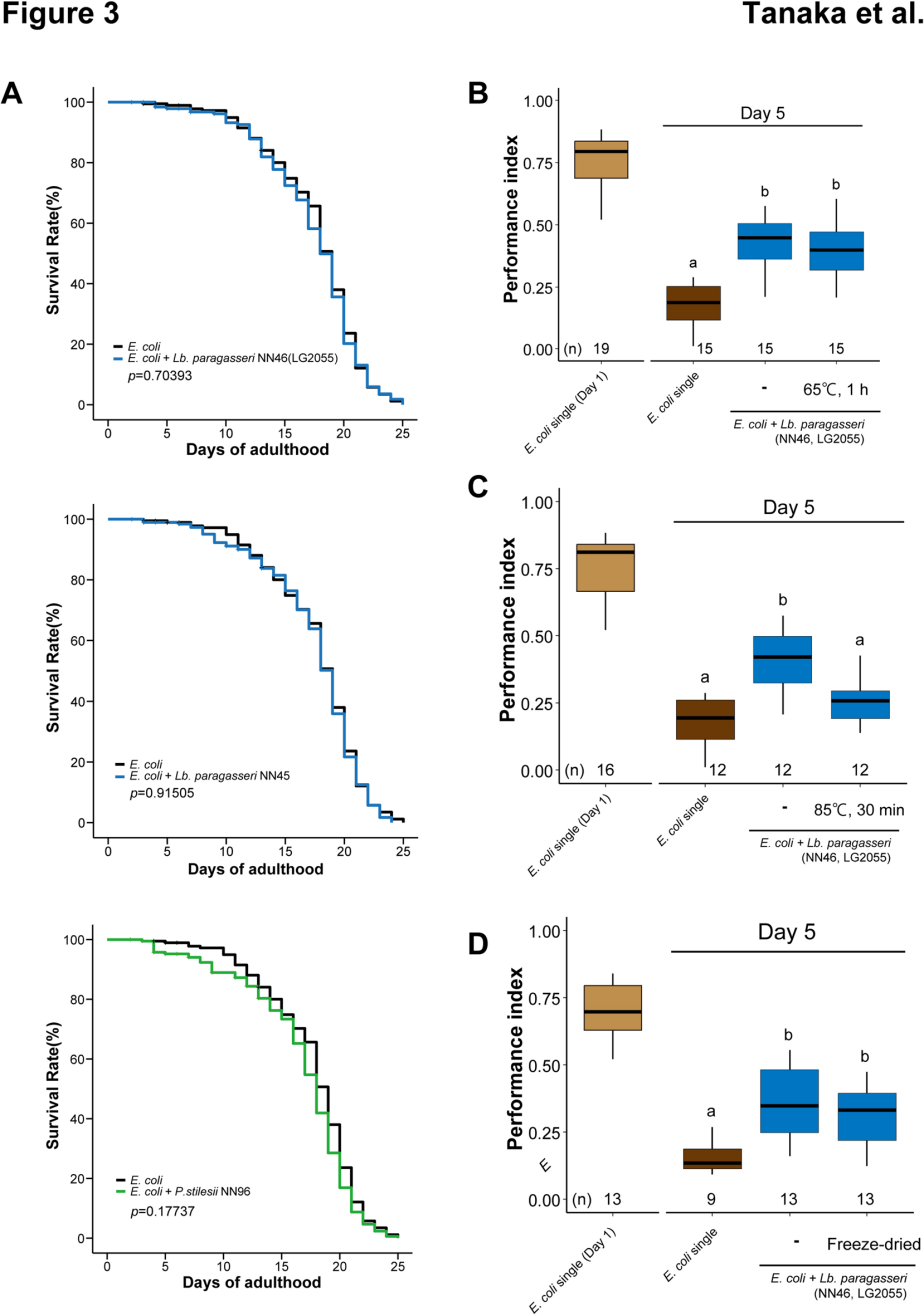
Ameliorative effect of *Lb. paragasseri* depends on heat-labile factors. **(A)** Lifespan of animals fed the indicated bacteria. The control *E. coli*-fed data were identical. Log-rank test was used for statistical analysis, adjusted with the Benjamini-Hochberg method. **(B-D)** Thermotaxis performance indices of animals fed *Lb. paragasseri* that were subjected to different treatments before mixing with *E. coli* at a 2:1 ratio. **(B)** 65 °C for 1 h. **(C)** 85 °C for 30 min. **(D)** Freeze-drying. Statistics: Median indices labeled with distinct letters are significantly different (*p* < 0.05) according to Kruskal-Wallis/Steel-Dwass tests.

We next investigated how LAB influence age-dependent thermotaxis decline. Some LAB are known to extend *C. elegans* lifespan^29–32^. By contrast, monoxenic feeding with *Lm. reuteri* (NN67) affects the age-dependent thermotaxis decline without affecting the lifespan^25^. Thus, we asked whether the ameliorative effect of LAB on thermotaxis results from lifespan extension. For the lifespan assay, we used peptone-free NGM plates to avoid unnecessary growth of *E. coli*. The absence of the peptone did not affect the effect of *Lb. paragasseri* (NN45, NN46) and *P. stilesii* (NN96) on thermotaxis (Supplementary Fig. 3). Animals fed a mixture of *E. coli* and *Lb. paragasseri* (NN45 or NN46) had lifespans similar to those fed *E. coli* (Fig. 3A). Animals fed a mixture of *E. coli* and *P. stilesii* showed a trend toward shortened lifespan, although the effect was not statistically significant (Fig. 3A). These results indicate that *Lb. paragasseri* and *P. stilesii* ameliorate age-dependent thermotaxis decline independently of lifespan regulation.

To further investigate the molecular and neuronal mechanisms underlying the ameliorative effect of LAB, we focused on *Lb. paragasseri* (NN46), which strongly improved thermotaxis without affecting body size, fat storage, or lifespan. Notably, *Lb. paragasseri* (NN46) was previously reported to extend *C. elegans* lifespan when provided alone (not mixed with *E. coli*)^29^. Since the previous study described NN46 as LG2055 or SBT2055, we refer to it here as *Lb. paragasseri* (NN46, LG2055). In thermotaxis, *Lb. paragasseri* (NN46) feeding without *E. coli* did not further enhance its effect as a mixture with *E. coli* (Supplementary Fig. 4A). Furthermore, cultivating animals with the mixture of *E. coli* and *Lb. paragasseri* (NN46, LG2055) from eggs did not affect the thermotaxis at Day 1, suggesting the ameliorative effect of *Lb. paragasseri* (Supplementary Fig. 4B). *Lb. paragasseri* (NN46, LG2055) can affect *C. elegans* physiology either as nutrition or as live bacteria. To address whether the live bacteria are required for the effect, we heat-killed *Lb. paragasseri* at 65 °C for 1 h, which reduced the bacterial colony-forming units (CFU) from 10^9^ CFU/mL to an undetectable level (see Materials and Methods). This heat-killed *Lb. paragasseri* retained the ability to improve the thermotaxis performance of Day 5 animals (Fig. 3B). To assess the thermostability of the ameliorative factor, we treated *Lb. paragasseri* at high and low temperatures. Heating at 85 °C for 30 min decreased the ameliorative effect, whereas freeze-drying did not affect it (Fig. 3C and D), indicating that the active factor in *Lb. paragasseri* is heat-labile but resistant to freezing.

### *Lb. paragasseri* suppresses AWC hyperactivity in aged animals

**Fig. 4:**
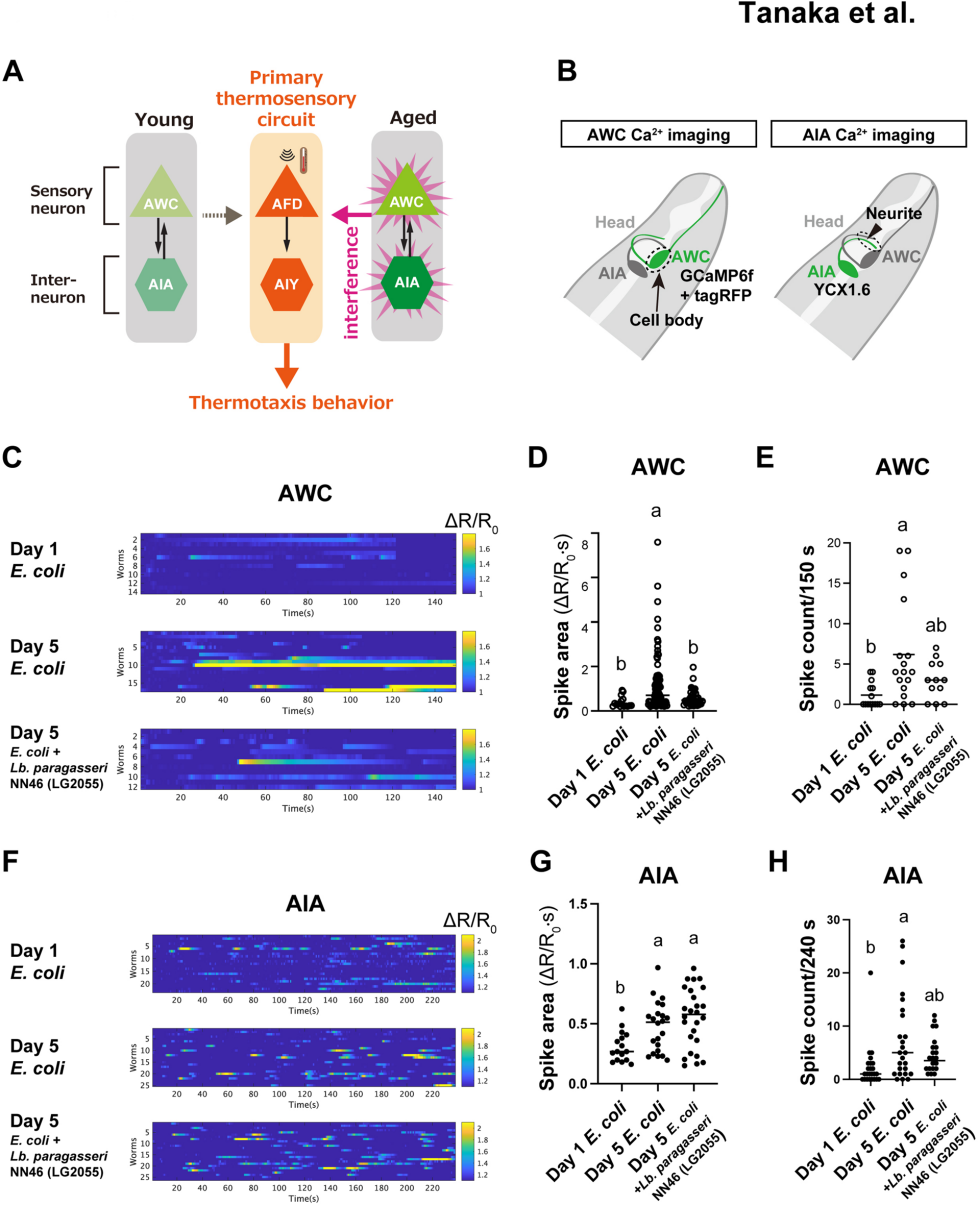
*Lb. paragasseri* suppresses hyperactivation of AWC neurons in aged animals. **(A)** Model of age-dependent thermotaxis decline in *E. coli*-fed animals. In aged animals, the primary thermosensory circuit (consisting of AFD thermosensory neurons and AIY interneurons) remains relatively intact, but AWC sensory neurons and AIA interneurons in aged animals are hyperactive, interfering with the primary circuit and causing thermotaxis defects. **(B)** Schematic of AWC sensory neurons and AIA interneurons in the head of an animal. Ca^2+^ activity of AWC and AIA was measured in the soma (arrow) and the neurite (arrowhead), respectively. **(C-H)** The quantification of Ca^2+^ activity in AWC or AIA neurons from *E. coli*-fed Day 1, *E. coli*-fed Day 5, and *E. coli* + *Lb. paragasseri*-fed Day 5 animals. **(C and F)** Heat map of calcium activity in AWC (C) and AIA (F) without temperature stimulus. Each line represents a single neuron recording. **(D**,** E**,** G and H)** Spike analyses of (C) and (F). **(D and G)** Area of calcium spikes in AWC (D) and AIA (G) neurons. Each dot represents an individual spike. The data from multiple neurons were combined. **(E and H)** Frequency of calcium spikes in AWC (E) and AIA (H). Each dot represents an individual neuron. Statistics: Tukey’s multiple comparison test was used to compare all the pairs. Different letters indicate statistical difference (*p* < 0.05).

We previously showed that age-dependent thermotaxis decline in *E. coli*-fed animals is caused by aberrant hyperactivity of AWC sensory neurons and AIA interneurons^26^(Fig. 4A). In our model, hyperactive AWC and AIA in aged animals interfere with the intact primary thermosensory circuit consisting of AFD and AIY neurons. To investigate the neuronal basis of how *Lb. paragasseri* (NN46, LG2055) can ameliorate age-dependent thermotaxis decline, we measured neuronal activity in AWC soma and AIA neurite using the Ca^2+^ indicators, GCaMP6f and YCX1.6, respectively^33,34^(Fig. 4B-H). AWC sensory neurons in *E. coli-*fed Day 5 animals showed spontaneous hyperactivity even without temperature stimuli, compared to Day 1 animals (Fig. 4C). Quantification of the calcium spikes confirmed that *E. coli*-fed Day 5 animals had larger spike area and higher frequency than Day 1 animals (Fig. 4D and E). Feeding with *Lb. paragasseri* decreased the AWC calcium spike area and frequency in Day 5 animals (Fig. 4C-E). AWC sensory neurons form bidirectional synapses with AIA interneurons (Fig. 4A), and the communication between these neurons, through neurotransmitters and neuropeptides, contributes to their hyperactivity^26^. Consistent with AWC results, AIA interneurons in the *E. coli*-fed Day 5 animals were hyperactive, with larger spike area and higher frequency than in Day 1 animals (Fig. 4F-H). Unlike its effect on AWC, *Lb. paragasseri* did not significantly alter AIA spike area or frequency in Day 5 animals (Fig. 4F-H). Together, these findings suggest that the neuronal basis of the ameliorative effect of *Lb. paragasseri* is associated with the suppression of aberrant hyperactivation of AWC sensory neurons in aged animals.

### Molecular mechanism underlying the effect of *Lb. paragasseri* on age-dependent thermotaxis decline

**Fig. 5:**
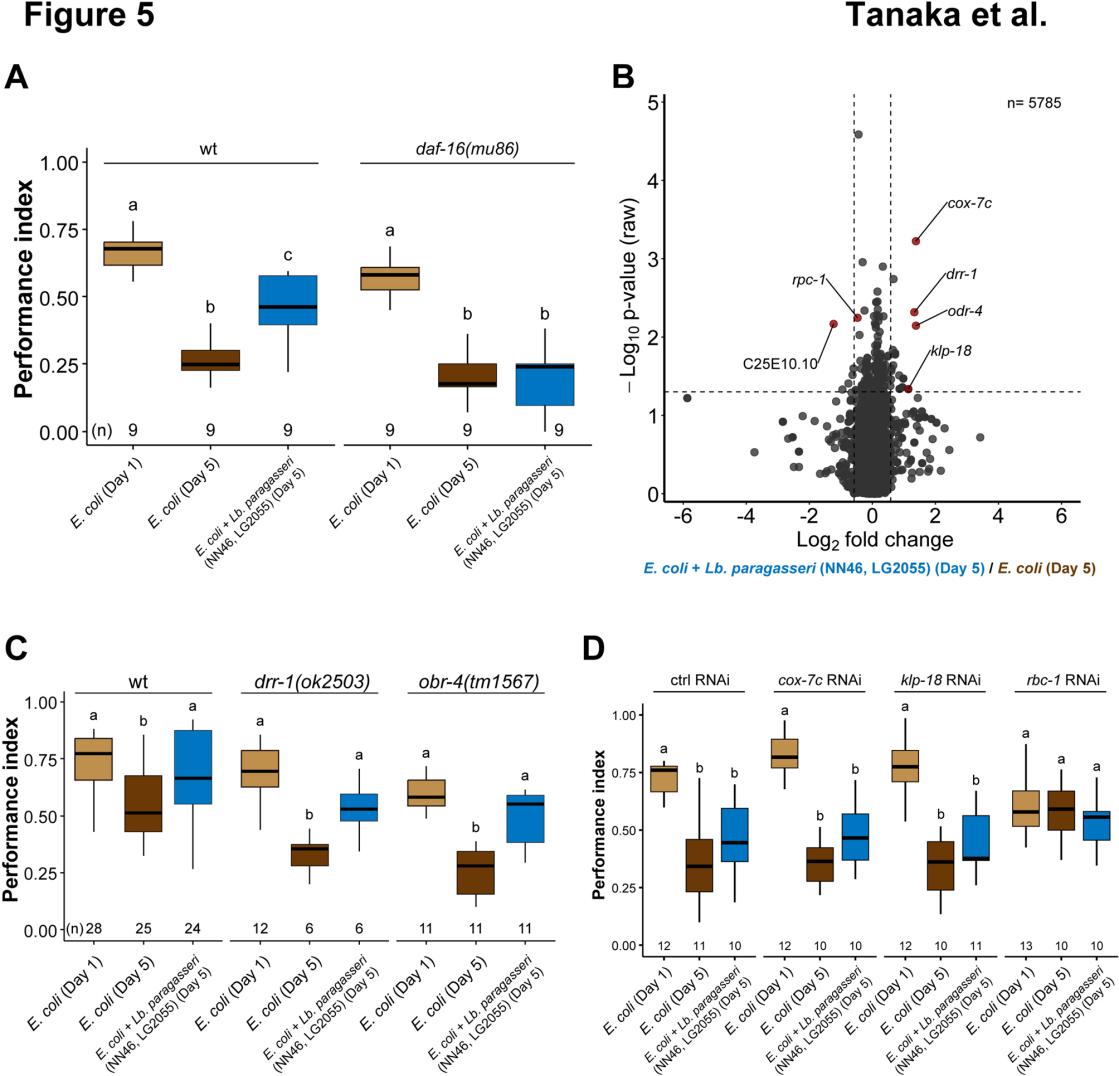
Genetic and proteomic analyses of the effects of *Lb. paragasseri.* **(A)** Effect of the *daf-16* mutation on the ameliorative effect of *Lb. paragasseri*. Thermotaxis performance indices are shown for the indicated ages and feeding conditions in wild-type and *daf-16(mu86)* mutants. **(B)** Volcano plot of the comparative proteome analysis between *E. coli*-fed and *E. coli* + *Lb. paragasseri*-fed aged animals. **(C)** Thermotaxis performance indices of the indicated mutants and feeding conditions. **(D)** Thermotaxis performance indices of Day 1 and Day 5 animals treated with control or indicated RNAi bacteria with or without *E. coli* from the L4 stage. Statistics: The mean indices labeled with distinct letters are significantly different (*p* < 0.05) according to Kruskal-Wallis, followed by Steel-Dwass test, within the same genotype or RNAi condition.

Our previous report has shown that the ameliorative effect of monoxenic culture with *Lm. reuteri* on aged animals requires *daf-16*, which encodes the homolog of the FOXO (Forkhead Box O) transcription factor in mammals^25^. We investigated whether the *daf-16* pathway also contributes to the effect of *Lb. paragasseri* (NN46, LG2055). The beneficial effect of feeding with the mixture of *E. coli* and *Lb. paragasseri* was abolished in the *daf-16(mu86)* null mutants, indicating the requirement of *daf-16* (Fig. 5A).

To comprehensively identify the proteins altered by *Lb. paragasseri* feeding, we performed proteomic analysis of Day 5 animals fed *E. coli* alone and those fed the mixture of *E. coli* and *Lb. paragasseri* (NN46, LG2055). Among the 5,785 proteins identified, 16 proteins were significantly upregulated, and 5 proteins were significantly downregulated by *Lb. paragasseri* (NN46, LG2055) supplementation (raw *p* < 0.05, > 1.5-fold change, Fig. 5B, Supplementary Fig. 5A and B). Top three upregulated genes were *cox-7c* (*c*ytochrome *ox*idase assembly protein), which encodes cytochrome c oxidase subunit 7 C involved in mitochondrial electron transport^35^; *drr-1* (*d*ietary *r*estriction *r*esponse), which is involved in lifespan modulation during dietary restriction^36^; *obr-4* (*o*xysterol *b*inding protein (OSBP) *r*elated), which is involved in cholesterol transport^37,38^. To elucidate the biological functions of proteins altered by *Lb. paragasseri* (NN46, LG2055) feeding, we performed Gene Ontology (GO) enrichment analysis on the differentially expressed proteins described above (THE GENE ONTOLOGY RESOURCE: https://geneontology.org/), but did not find any significant enrichment in any categories—Cellular Component (CC), Biological Process (BP), or Molecular Function (MF)—for either 16 upregulated or 5 downregulated proteins (Supplementary Fig. 5A and B).

To determine whether candidate genes identified from the proteomic analysis contribute to the amelioration of age-dependent thermotaxis decline, we examined loss-of-function mutants of these genes in *C. elegans*. From the significantly increased proteins, we prioritized candidates with available mutants and examined thermotaxis performance in the following mutants. In *drr-1* and *obr-4* mutants, *Lb. paragasseri* still improved thermotaxis, suggesting that these genes are not essential for the ameliorative effect (Fig. 5C). Among candidates whose mutants were not available, we performed RNA interference (RNAi) experiments to suppress the function of candidate genes. We tested *cox-7c* (described above); *klp-18* (*k*inesin-*l*ike *p*rotein), which encodes a kinesin motor protein involved in spindle assembly during meiosis^39^; and *rbc-1* (*r*a*bc*onnectin related), which is predicted to be part of the RAVE complex involved in vacuolar acidification (WormBase). Animals were fed dsRNA-expressing *E. coli* (HT115) instead of the regular *E. coli* (OP50), or a mixture of dsRNA-expressing *E. coli* and *Lb. paragasseri* (NN46, LG2055) from the last larval stage (fourth larval stage, L4) onward. Under RNAi conditions, *Lb. paragasseri* (NN46) showed a modest improvement in thermotaxis of Day 5 animals fed control bacteria expressing the empty vector, but the effect was not statistically significant (Fig. 5D, ctrl RNAi). RNAi-mediated knockdown of *cox-7c* and *klp-18* showed a similar trend of amelioration by *Lb. paragasseri* (NN46, LG2055), suggesting that these genes may not be involved in the effect of *Lb. paragasseri* (Fig. 5D). On the other hand, *rbc-1* RNAi increased the thermotaxis ability of *E. coli*-fed aged animals, masking the beneficial effect of NN46. *rbc-1* RNAi also slightly impaired thermotaxis in the young animals, indicating that *rbc-1* is required for normal thermotaxis. These results suggest that *rbc-1* does not specifically mediate the age-dependent beneficial effects of NN46, but rather plays a role in thermotaxis. Together, our results imply that multiple *daf-16*-regulated genes may collectively contribute to the ameliorative effect of *Lb. paragasseri* (NN46, LG2055).

### Discussion

Different bacterial diets affect *C. elegans* behaviors. Our previous study implied that dietary *E. coli* causes age-dependent thermotaxis decline, rather than *Lm. reuteri* containing factors that actively ameliorate it^25^. This conclusion was based on two observations: (1) the deteriorative effect of *E. coli* dominates, as feeding animals the mixture of *E. coli* and *Lm. reuteri* (1:2 ratio) caused thermotaxis decline; (2) heat treatment of *E. coli* ameliorated the thermotaxis decline^25^, suggesting that *E. coli* contains heat-sensitive deteriorative factors. Given that we could not find any bacteria that support the growth of animals and prevent the age-dependent thermotaxis decline^25^, overall nutrition from *E. coli*, instead of specific factors, may cause the decline. In contrast to *Lm. reuteri*, however, *Lb. paragasseri* showed ameliorative effects even when mixed with *E. coli*. Therefore, *Lb. paragasseri* may contain factors that counteract the deteriorative effects of *E. coli*. Consistent with this notion, heat treatment abolished the ameliorative effect of *Lb. paragasseri* (NN46, LG2055), implying that heat-labile factors mediate the effect. These factors may be bacterial species-specific rather than strain-specific, since another strain (NN45) of *Lb. paragasseri* showed similar ameliorative properties to NN46 (LG2055).

Major components of bacteria are the cell wall (peptidoglycan, teichoic acids, and lipoteichoic acids), the cell membrane (fatty acids, phospholipids, glycolipids, etc.), proteins, and nucleic acids^40–42^. Because the ameliorative effect was observed with heat-killed NN46 (LG2055), bacterial components rather than living bacteria likely contribute to the observed effects. *E. coli* and lactic acid bacteria are classified as Gram-negative and Gram-positive bacteria, respectively, and their cell envelope structures differ substantially. Gram-negative bacteria have a thin peptidoglycan layer and an outer membrane. In contrast, Gram-positive bacteria have a thick peptidoglycan layer that contains teichoic and lipoteichoic acids and lack an outer membrane^40,41,43^. Even within lactic acid bacteria, cell wall composition and structure are reported to vary^41^. Analyzing variation in these components may help identify key beneficial factors.

*Lb. paragasseri* (NN46, LG2055) was initially classified under the *Lb. gasseri* species^44^. In *C. elegans*, previous reports demonstrated that monoxenic culture with *Lb. paragasseri* (LG2055) extends lifespan^29^. In our assays, *Lb. paragasseri* (LG2055) provided as a mixture with *E. coli* did not extend the lifespan, even though it preserved thermotaxis in aged animals. Thus, the physiological outcomes of *Lb. paragasseri* (LG2055) appear context-dependent: lifespan extension requires monoxenic culture, whereas thermotaxis preservation occurs even in the presence of *E. coli*. The strain dependence of lifespan extension further underscores this complexity, as *Lb. paragasseri* (LG2055) prolongs lifespan more strongly than *Lb. gasseri* (JCM1131^T^, type strain)^29^.

Previous work showed that lifespan extension by monoxenic culture of *Lb. paragasseri* (LG2055) depends on activation of SKN-1, which encodes a basic leucine zipper transcription factor through the p38 MAPK pathway, but does not depend on the DAF-16 transcription factor^29. ^ By contrast, we found that the beneficial effect of *Lb. paragasseri* (LG2055) for the thermotaxis preservation requires DAF-16. Taken together, these findings imply that *Lb. paragasseri* (LG2055) can act through distinct pathways depending on the dietary regimen. Proteome analysis revealed a modest number of significantly altered proteins following *Lb. paragasseri* (NN46, LG2055) supplementation. This is consistent with the observation that *Lb. paragasseri* supplementation did not substantially affect gross physiological traits such as body length, size, lipid accumulation, or lifespan. Instead of a substantial change in many proteins, the benefits of *Lb. paragasseri* may arise from small adjustments in various proteins regulated by the DAF-16 transcription factor. Indeed, in mammals, *Lb. paragasseri* (NN46, LG2055) affects various physiological aspects such as reducing obesity^45^, alleviating inflammation^46^, improving glucose metabolism^47^, and boosting immunity^48,49^. Because the beneficial effect of *Lb. paragasseri* (LG2055) is abolished by heat treatment at 85 °C, investigating proteins upregulated by *Lb. paragasseri* (LG2055) may help identify crucial factors for ameliorating the age-dependent thermotaxis decline.

Previously, we demonstrated that the hyperactivation of AWC sensory neurons and AIA interneurons in aged nematodes contributes to thermotaxis decline^26^. Furthermore, we showed that the overactivation of AWC due to aging is suppressed by monoxenic culture with *Lm. reuteri*, which concurrently mitigates thermotaxis decline. These findings indicate that age-dependent changes in neurons and neural circuits can be modulated by diet. When *Lb. paragasseri* (LG2055) was provided in combination with *E. coli*, AWC activity was reduced. Although the calming effects on AWC and AIA were stronger with monoxenic *Lm. reuteri* culture, the mild calming effect of *Lb. paragasseri* (LG2055) on AWC may also contribute to suppressing age-dependent thermotaxis decline. The neuromodulatory role of LAB has been observed across species. In *C. elegans*, *Lb. rhamnosus* Probio-M9 improved locomotor ability and slowed the decline in muscle function^31^. These effects are associated with enhanced stress resistance and activation of mitochondrial unfolded protein response pathways^31^. In mice, ingestion of *Lb. rhamnosus* JB-1 reduced stress-induced corticosterone levels and altered GABA receptor expression in the brain, resulting in reduced anxiety- and depression-related behaviors^50^. Similarly, *Lb. plantarum* PS128 alleviates anxiety- and depression-like behaviors in rodent models via modulation of dopamine and serotonin pathways^51^. In humans, randomized clinical trials report that *Lb. helveticus* R0052 and *B. longum* R0175 supplementation reduces psychological distress and improves mood^52^. These findings suggest that LAB effects extend beyond metabolic or lifespan traits to include neural circuit modulation, stress resilience, and mood regulation.

In sum, our findings indicate that *Lb. paragasseri* produces heat-labile factors that can override *E. coli*–induced deterioration of thermotaxis in aged animals. The effect depends on DAF-16 and appears mechanistically distinct from the SKN-1–mediated lifespan extension observed under monoxenic *Lb. paragasseri* (LG2055) feeding. This result highlights the importance of dietary context in shaping host–microbe interactions and suggests that *Lb. paragasseri* may influence aging phenotypes via multiple, pathway-specific mechanisms. Together with evidence from nematodes, rodents, and humans, our findings highlight LAB as neuroactive modulators, shaping host behavior through metabolite-mediated signaling and host transcriptional pathways. Future identification of the heat-labile metabolites will be critical to elucidate how *Lb. paragasseri* exerts its protective effects.

## Materials and methods

### *C. elegans* and bacterial strains

*Caenorhabditis elegans* N2 (Bristol) was used as the wild-type strain. CF1038 *daf-16 (mu86)*, RB1924 *drr-1(ok2503)*, FX1567 *odr-4(tm1567)*, and FX8515 C25E10.1*(tm8515)* were used for the thermotaxis assays. *C. elegans* strains were maintained at 23 °C on Nematode Growth Medium (NGM) plates seeded with *E. coli* OP50, as previously described^11^. Synchronized eggs were prepared for all the experiments by bleaching gravid adults using a solution containing a 1:1 ratio of household bleach and 1 N NaOH. Animals cultured at 23 °C for 3 days after egg preparation were considered day 1 adults (Day 1) in this study. The bacterial strains used are listed in Supplementary Table 1.

### Bacterial plates

Bacterial strains were cultured under the conditions described in Supplementary Table 1. Bacterial cells were collected by centrifugation (7,000 g, 5–10 min, 4 °C), washed twice with sterile saline (0.9% NaCl), and resuspended in NG buffer (25 mM potassium phosphate buffer (pH 6.0), 50 mM NaCl, 1 mM CaCl_2_, 1 mM MgSO_4_) to 0.1 g/mL wet pellet for LAB (hereafter referred to as the untreated LAB suspension) and 0.05 g/mL wet pellet for *E. coli* OP50. For heat-killed preparations, the untreated LAB suspensions were heated at 65 °C for 1 h or 85 °C for 30 min, resulting in < 10 CFU/mL. To prepare LAB powder, the collected and washed bacterial cell pellet was suspended in sterile water and freeze-dried. After determining the total bacterial cell counts (including both live and dead cells) in the LAB powder and in the untreated LAB suspension, the LAB powder was resuspended in NG buffer and adjusted so that the total bacterial cell counts matched those of the untreated LAB suspension. The total bacterial cell counts (cells/g powder and cells/mL suspension) were determined using a hemocytometer. For the powder, a known amount of powder was suspended in a defined volume of sterile water, counted with a hemocytometer, and converted to cells/g. For mixed cultures, LAB and *E. coli* OP50 suspensions were mixed at a 1:1 volume ratio (corresponding to a 2:1 bacterial concentration, which was chosen to maximize the potential effect of LAB for the screen while maintaining the normal growth of animals by feeding *E. coli*). Two hundred microliters of suspension were spread onto 55 mm-diameter NGM plates. Control plates were prepared with 200 µL of *E. coli* OP50 suspension alone.

### Preparation of aged animals

Synchronized Day 1 adults were collected and washed in NG buffer and transferred daily to new plates under the designated bacterial conditions. This process was repeated daily for 4 days to obtain Day 5 adults. Newly laid eggs and larvae were removed during daily transfers.

### Thermotaxis assay

Thermotaxis assays were performed as previously described^25^(Fig. 1B). A linear temperature gradient (~ 0.5 °C/cm) was established on a custom-made metal bridge. Fifty to 200 animals cultivated at 23 °C were collected, washed in NG buffer, and placed at the center (20 °C) of a rectangular agar plate (14 × 10 cm, containing 20 g of Bacto Agar (BD), 3 g of NaCl, and 25 mL of 1 M potassium phosphate buffer in 1 L of H_2_O) on the metal bridge with a 17–23 °C gradient without food. After 1 h, the animals were killed with chloroform and counted. The performance index was calculated according to the formula in Fig. 1B. In our experimental setting, it is the fraction of animals present in Sects. 7 and 8 divided by the total number of animals.

### Lifespan assay

Animals were synchronized by bleaching and cultivated on OP50-seeded NGM plates until Day 1. Day 1 adults were washed three times with NG buffer and transferred to peptone-free NGM plates seeded with either OP50 or the indicated bacterial mixtures. Animals were transferred to fresh bacterial plates daily until Day 5. Subsequently, animals were transferred every other day, and survival was scored. Death was defined as the absence of response to head and tail touches. Each group consisted of 100 animals (25 animals/plate, 4 plates per group).

### Analysis of growth and lipid accumulation

Day 5 animals fed *E. coli* OP50, *Lm. reuteri*, or bacterial mixtures were collected and washed in PBS-T (1% Triton X-100 in Phosphate-Buffered Saline), and fixed in 40% isopropanol. After removing the supernatant, animals were stained with the 0.5% Oil Red O solution prepared in isopropanol for 2 h, followed by gentle rotation in PBS-T for 30 min. The stained animals were transferred onto 4% agar pads and imaged using AxioImager A2 equipped with a color CCD camera and LED light source (Colibri 7) (Zeiss). Oil Red O staining intensity was quantified using ImageJ software^53^.

### Calcium imaging

Animals for neural activity measurements were prepared as described above. For calcium imaging, we used transgenic *C. elegans* strains expressing the calcium indicator GCaMP6f^33^ in AWC neurons or YCX1.6 ^34^ in AIA neurons. For AWC imaging, tagRFP, which fluoresces independently of calcium, was co-expressed to provide a reference signal for normalization of GCaMP6f fluorescence. Specifically, strain IK3296 *njEx1368[ceh-36p::GCaMP6f str-2p::tagRFP]* was used to image AWC sensory neurons, and strain IK3331 *njEx1387[ins-1p::FLP + gcy-28dp::FRT::stop::FRT::stop::YCX1.6]* to image AIA interneurons. Calcium imaging was performed as previously described^26^. A thin 10% agar pad was placed on a 24 mm x 24 mm cover glass, onto which 2 µL of polystyrene beads were spotted. A single worm was transferred from the cultivated NGM plate onto the agar pad and covered with a circular coverslip for immobilization. After placing the worm on the imaging stage, imaging was initiated immediately after a 2-min waiting period. To measure the spontaneous activity, the temperature was precisely maintained at 22.5 °C by a Peltier-based temperature controller. Neuronal activity was then recorded at 1 frame per second (fps) for 150 s in AWC neurons and for 240 s in AIA neurons. Fluorescence intensities of calcium indicators and tagRFP were analyzed using ImageJ. For each sample, the GCaMP6f/tagRFP or YFP/CFP ratio (R) was calculated, and neural activity was normalized by dividing the time series by the mean fluorescence intensity during the initial 0–10 s period (R_0_). Heatmaps were generated to visualize normalized activity as ΔR/R_0_, in which brighter colors indicate stronger activity. To compare the neuronal activity and spike frequency across strains, calcium-spike deconvolution and denoising were performed using a custom MATLAB (MathWorks) script^26,54^. Spikes with a peak threshold of ≥ 0.2 for AWC and ≥ 0.15 for AIA were defined as activity events, and adjacent spikes with a spacing of ≤ 3 frames were merged into a single event. Spike frequency was calculated as the number of inferred spike events per recorded time (count/150 s for AWC or count/240 s for AIA). Spike area (ΔR_0_/R·s) was quantified as the area under each inferred spike event, reflecting the magnitude of neuronal activity.

### Sample preparation for Liquid Chromatography-Tandem Mass Spectrometry (LC-MS/MS)

Day 5 animals fed *E. coli* or *E. coli + Lb. paragasseri* (LG2055) were collected (300–500 animals per sample) into tubes containing NG buffer. After three washes, animals were incubated in NG buffer for 20 min to remove intestinal contents. The supernatant was removed, and the animals were washed with sterile water. After removing the supernatant, the samples were stored at -80 °C until use. Proteins were extracted from frozen animals in 100 mM Tris-HCl (pH 8.5) containing 4% sodium dodecyl sulfate (SDS) and 20 mM NaCl by sonication using a BIORUPTOR BR-II (SONIC BIO Co., Kanagawa, Japan) set to “High” with 30 s on/off cycles for 10 min. Lysates were centrifuged at 15,000 g for 15 min at 4 °C. Supernatants were collected and transferred to a fresh tube. The protein concentrations were determined using the Pierce™ 660 nm Protein Assay Kit with Ionic Detergent Compatibility Reagent (Thermo Fisher Scientific). Extracts were reduced with 25 mM dithiothreitol for 30 min at 25 °C and alkylated with 50 mM iodoacetamide for 30 min at 25 °C, in the dark. Protein purification and digestion were carried out using the single-pot, solid-phase-enhanced sample-preparation (SP3) method^55^. Beads were resuspended in 100 µL of 50 mM Tris-HCl (pH 8.0) containing 1 µg of Lys-C (Fujifilm-Wako) and incubated at 37 °C for 4 h, followed by digestion with 1 µg of trypsin (Promega) overnight at 37 °C with gentle mixing. Digested peptides were acidified with 5 µL of 20% trifluoroacetic acid (TFA), desalted using GL-Tip SDB (GL Sciences, Tokyo, Japan), evaporated in a SpeedVac concentrator, and redissolved in 0.1% TFA and 2% acetonitrile.

### Mass spectrometry analysis

Digested peptides were analyzed by nanoflow reversed-phase LC followed by tandem MS using a Q-Exactive hybrid mass spectrometer (Thermo Fisher Scientific) equipped with a nano-electrospray ionization (NSI) source (AMR). The capillary reversed-phase HPLC–MS/MS was composed of a Dionex U3000 gradient pump equipped with a VICI CHEMINERT valve. Desalted peptides were loaded into a separation capillary C18 reverse-phase column (NTCC-360/100–3–125, 125 × 0.1 mm, Nikkyo Technos). Peptide spectra were acquired across an m/z range of 350–1,800 using Xcalibur 4.1.50 (Thermo Fisher Scientific). Repeatedly, MS spectra were recorded, followed by 20 data-dependent high-energy collisional dissociation (HCD) MS/MS spectra generated from the 10 highest intensity precursor ions. MS/MS spectra were analyzed, and peak lists were generated using Proteome Discoverer 2.4.1.15 (Thermo Fisher Scientific). Database searches were performed with SEQUEST software package (Thermo Fisher Scientific) against the *Caenorhabditis elegans* proteome and the common Repository of Adventitious Proteins (cRAP) contaminant databases. Search parameters were as follows: enzyme specificity set to allow up to two missed cleavage sites, a mass tolerance of 10 ppm for peptide tolerance, 0.02 Da for MS/MS tolerance, fixed modification of carbamidomethyl (C), and variable modification of oxidation (M), phosphorylation (S, T, Y), and N-terminal acetylation. Peptides were identified based on significant Xcorr values using a high-confidence filter. Identified peptides were manually inspected to confirm peptide identities and modification assignments from the HCD MS/MS.

### Gene ontology analysis

Using data from mass spectrometry analysis, Gene Ontology (GO) analysis was performed on proteins differentially expressed between *E. coli*-fed and *E. coli* + *Lb. paragasseri* (LG2055)-fed Day 5 animals. To ensure accuracy, proteins identified as contaminants were excluded, and only those showing > 1.5-fold changes with raw *p* < 0.05 were analyzed. GO enrichment analysis was performed using the Gene Ontology Resource (https://geneontology.org/).

### RNA interference (RNAi) treatment

RNA interference by feeding was performed following a protocol similar to that described in previous studies^56,57^. The RNAi plates were prepared by supplementing peptone-free NGM agar with 5 mM IPTG and 100 µg/mL carbenicillin (final concentrations). Peptone-free plates were used to prevent *E. coli* growth during plate storage. The condition of the plates possibly weakened the effect of *Lb. paragasseri* (NN46, LG2055) (Fig. 5D, Ctrl RNAi). *E. coli* HT115 bacteria transformed with the L4440-based vector, expressing double-stranded RNA (dsRNA) targeting the genes of interest, were streaked onto ampicillin-containing plates. Single colonies were inoculated in Super Broth supplemented with 100 µg/mL ampicillin (final concentration) and cultured for 16 h at 37 °C with shaking. Bacteria were harvested by centrifugation at 6,000 rpm for 10 min at 4 °C, then washed twice with 0.9% NaCl. The cells were resuspended in NG buffer to a final concentration of 0.05 g/mL (wet weight). Two hundred microliters of bacterial suspension were spread onto 55-mm RNAi plates and dried overnight. For animal groups fed the bacterial mixture (*E. coli* + *Lb. paragasseri*), *Lb. paragasseri* was inoculated from frozen stock into MRS Broth and cultured for 16 h at 37 °C without shaking. Bacterial cells were harvested and resuspended at 0.1 g/mL in the same manner as *E. coli*. Equal volumes (100 µL each) of the two bacterial suspensions were mixed and spread onto RNAi plates. Synchronized L4-stage animals were washed off standard cultivation plates using NG buffer and transferred onto freshly seeded RNAi plates containing bacteria expressing dsRNA for each gene of interest. Animals were washed daily with NG buffer and transferred to fresh RNAi plates with bacteria until they reached Day 1 or Day 5 of adulthood. These animals were used for thermotaxis assays.

### Statistical analysis

Box plots indicate key summary statistics by showing the median, first and third quartiles, and data range. A line inside the box marks the median, while the bottom and top edges represent the first and third quartiles, respectively. The whiskers extend to the minimum and maximum values, excluding outliers, which are shown as individual points. To compare two groups, we employed either the Mann-Whitney U test or Student’s t-test, depending on the data distribution. When necessary, we adjusted the *p*-values using the Benjamini-Hochberg method to control the false discovery rate. For analyzing differences among multiple groups, the Kruskal-Wallis test, followed by Steel-Dwass test for post hoc analysis or ANOVA followed by Tukey multiple-comparison test, was used, as appropriate. Survival curves were compared using the log-rank test. Statistical analyses were conducted using R software (R Core Team, https://www.R-project.org/, Vienna, Austria), and the significance threshold was established at *p* < 0.05. Statistical comparisons of the amplitude and frequency of Ca^2+^ spikes were performed across age groups (Day 1 vs. Day 5) and feeding conditions (*E. coli* vs. *E. coli* + *Lb. paragasseri* (LG2055)) using GraphPad Prism. Significance was determined using Tukey’s multiple comparison test.

## Supplementary Information

Below is the link to the electronic supplementary material.

Supplementary Material 1

## Data Availability

Proteome data are available in ProteomeXchange (PXD071343) and jPOST (JPST004206)^58^. All other data generated during this study are included in the published article and its Supplementary Information files.
