# Supplementary material for: Lactobacillus paragasseri ameliorates age-dependent thermotaxis decline in Caenorhabditis elegans
Source: Sci Rep. 2026 Apr 17;16:17934. doi: 10.1038/s41598-026-48002-7 (PMC13249960; doi:10.1038/s41598-026-48002-7)
Supplement: Supplementary file 1 — Supplementary Material 1 [file 41598_2026_48002_MOESM1_ESM.pdf]

Supplementary Information

Lactobacillus paragasseri ameliorates age-dependent thermotaxis decline in *Caenorhabditis elegans*

Authors

Masaru Tanaka, Sachio Tsukada, Moon Sun Jang, Binta Maria Aleogho, Ikue Mori, and Kentaro Noma*

Figure S1

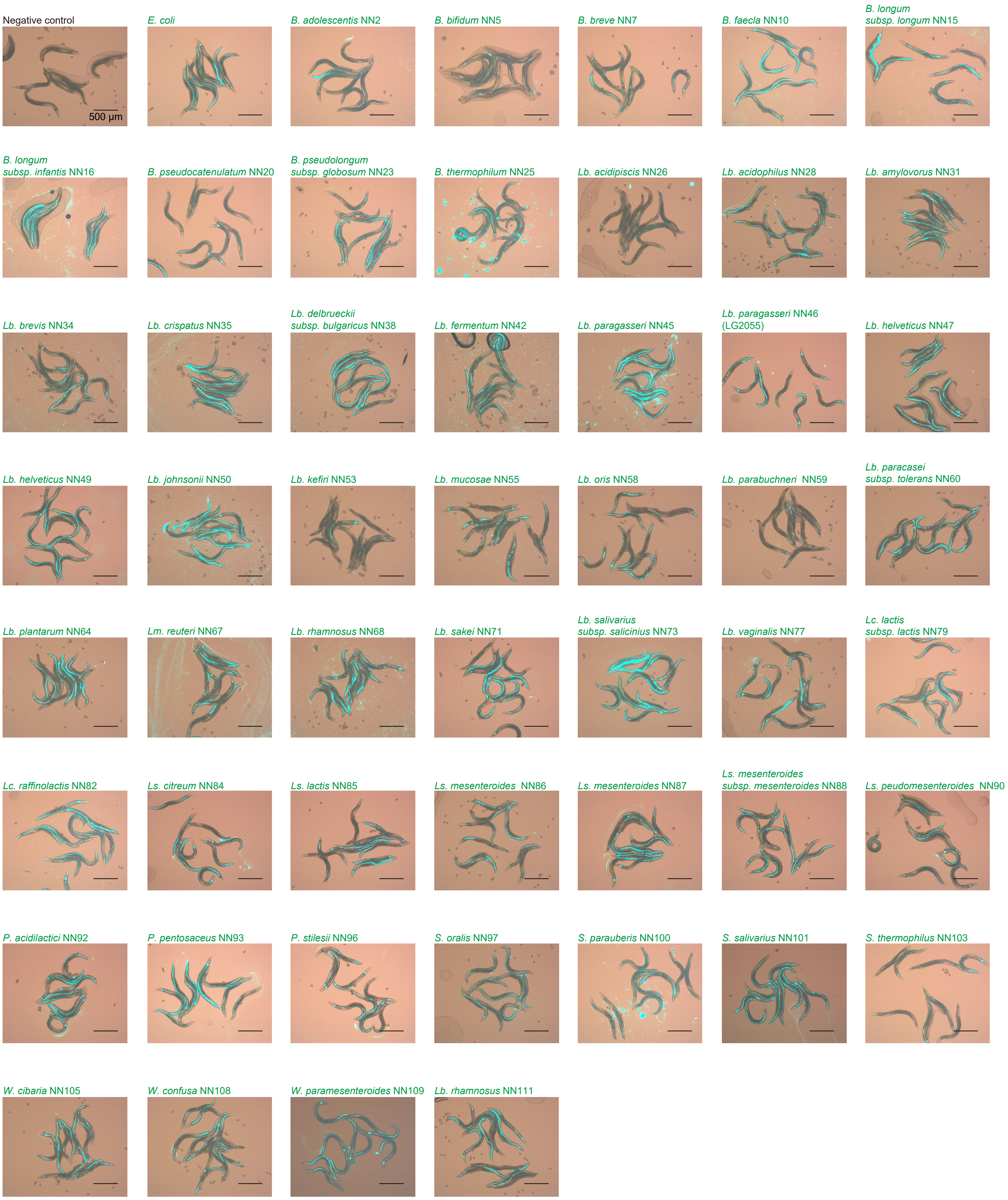

Supplementary Figure 1. LAB are ingested by *C. elegans*

Bacteria were fluorescently labeled with FITC and provided to Day 1 animals. Fluorescent images were taken after 24 h. Scale bar = 500 µm

Figure S2

A **Second screen**

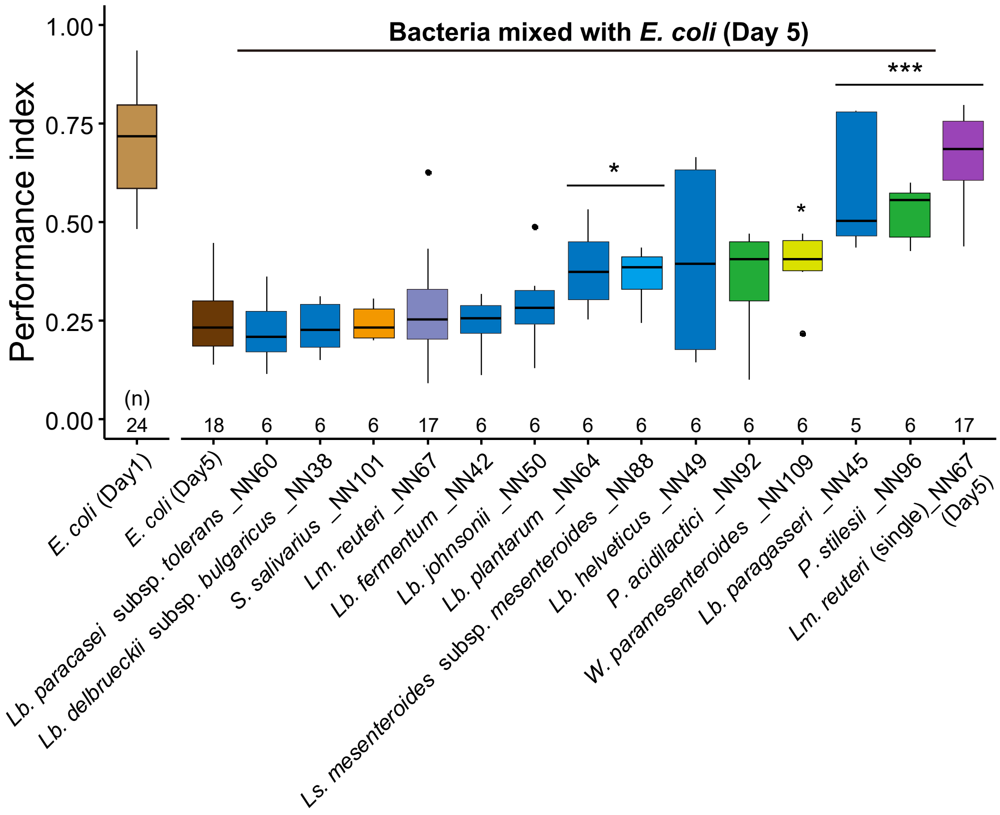

B **Third screen**

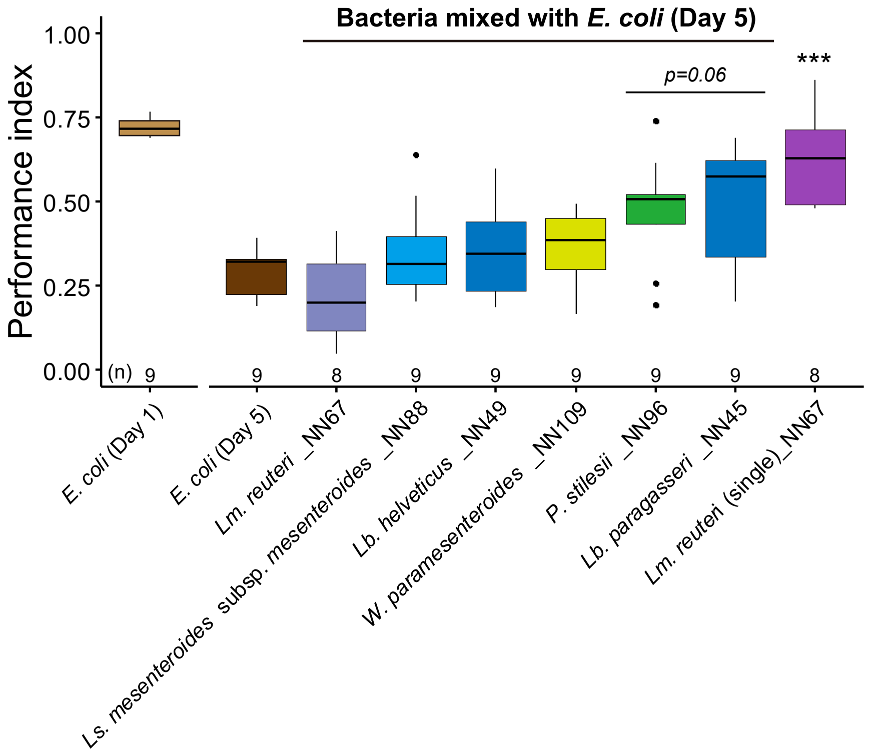

Supplementary Figure 2. Second and third screening of LAB for effects on thermotaxis in aged animals

Animals were fed a mixture of *E. coli* and the indicated LAB from Day 1 and assayed for thermotaxis at Day 5. Thermotaxis performance indices of the indicated ages and feeding conditions are shown for the second **(A)** and third **(B)** screening.

Figure S3

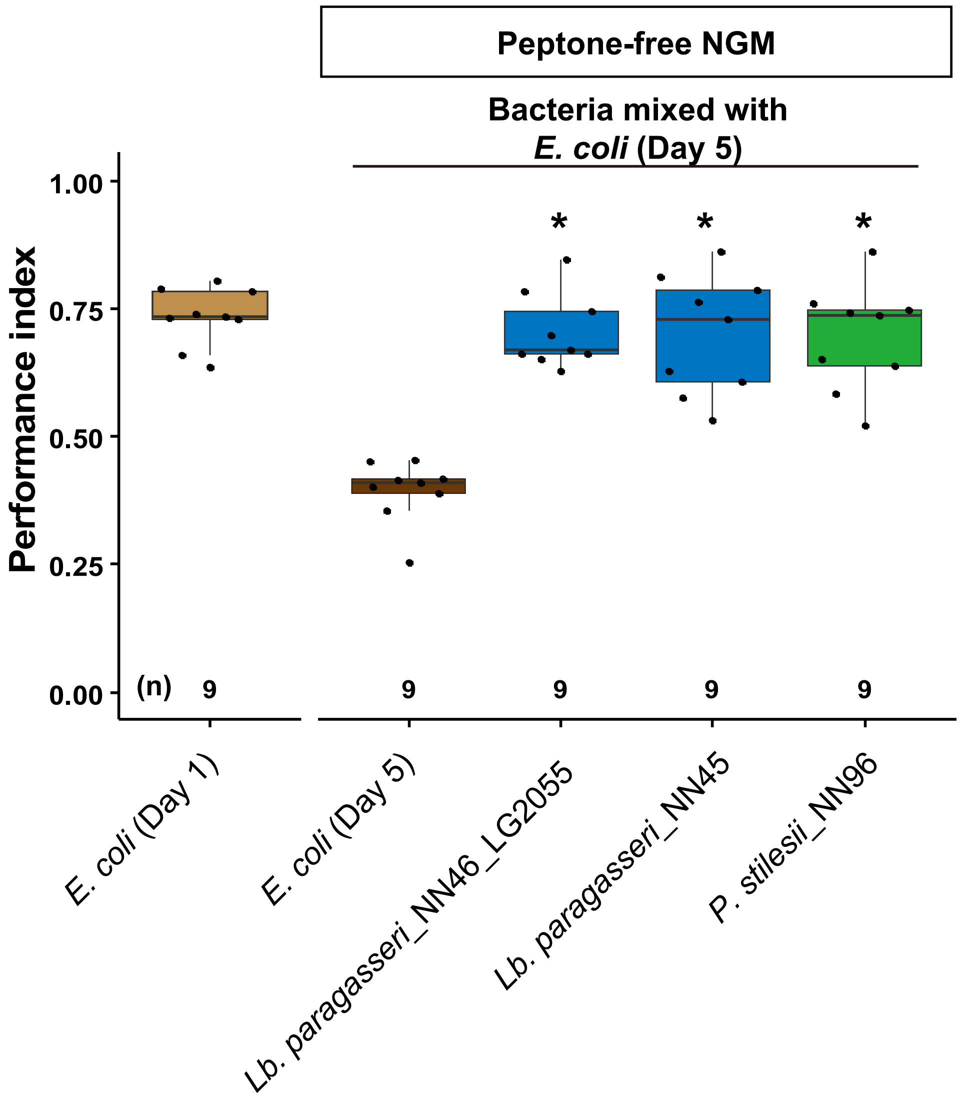

Supplementary Figure 3. The effect of peptone-free plates during aging on thermotaxis
Animals were grown on standard NGM plates until Day 1. On Day 1, they were transferred to peptone-free NGM plates with *E. coli* alone or a mixture of *E. coli* and the indicated LAB and assayed for thermotaxis at Day 5. Thermotaxis performance indices of the indicated ages and feeding conditions are shown.
Statistics: Mann–Whitney U test was used to compare each data with the *E. coli*-fed Day 5 condition and the p-values were adjusted using the Benjamini-Hochberg method. *$p < 0.05$.

Figure S4

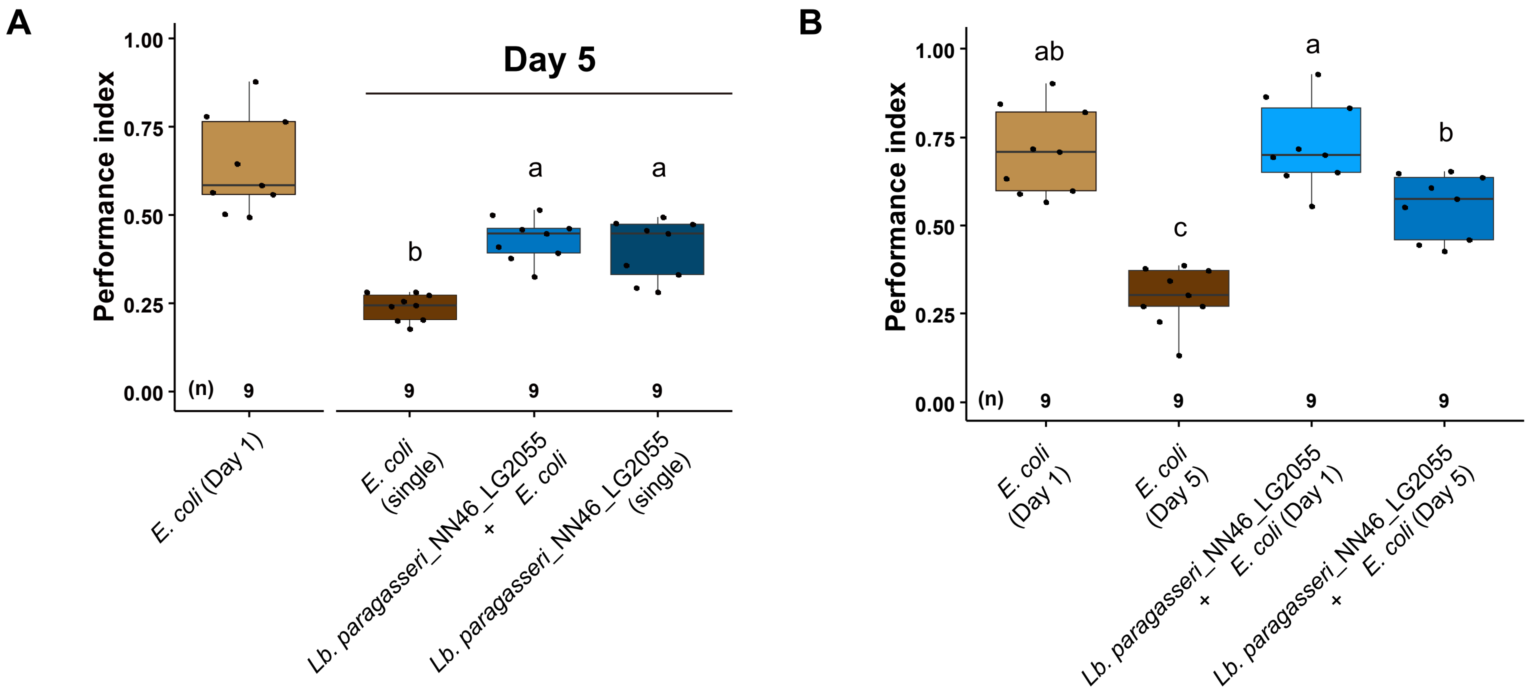

Supplementary Figure 4. The effect of *Lb. paragasseri* under different feeding regimens and administration onsets

(A) The effect of feeding *Lb. paragasseri* alone on thermotaxis. Animals were fed a *E. coli* alone, *Lb. paragasseri* alone or a mixture of *E. coli* and *Lb. paragasseri* from Day 1 and assayed for thermotaxis at Day 5. Thermotaxis performance indices of the indicated ages and feeding conditions are shown.

(B) Thermotaxis in young and aged animals fed a mixture of *E. coli* and *Lb. paragasseri* Animals were grown on a mixture of *E. coli* and *Lb. paragasseri* from egg stage and assayed for thermotaxis at Day5. Thermotaxis performance indices of the indicated ages and feeding conditions are shown.

Statistics: The mean indices labeled with distinct letters are significantly different ($p < 0.05$) according to Kruskal-Wallis, followed by Steel-Dwass test.

Figure S5

A

E. coli + Lb. paragasseri (NN46, LG2055) (Day 5) > E. coli (Day 5)

Accession	Protein description	Gene name	Sequence name	Fold change	p-valure(raw)
O18687	Cytochrome c oxidase polypeptide VIIc	*cox-7c*	F26E4.6	2.605	0.001
O01686	Oxysterol-binding protein	*obr-4*	C32F10.1	2.609	0.007
O02268	MARVEL domain-containing protein	*drr-1*	F45H10.4	2.52	0.005
G5EBX0	Kinesin motor domain-containing protein (Kinesin-like protein-18)	*klp-18*	C06G3.2	2.205	0.046
A0A4V0IKJ4	RAVE complex protein Rav1 C-terminal domain-containing protein	*rbc-1*	F54E4.1	1.974	0.034
Q10038	Tyrosine-protein phosphatase (Vh1 dual specificity phosphatase family protein 1)	*vhp-1*	F08B1.1	1.935	0.044
C6KRN3	Protein YIPF	W02D9.2	W02D9.2	1.819	0.046
O16517	ATP synthase-coupling factor 6, mitochondrial	*atp-4*	T05H4.12	1.801	0.031
O76410	NADH:flavin oxidoreductase/NADH oxidase N-terminal domain-containing protein	T10B5.8	T10B5.8	1.642	0.016
Q9TZ51	Fatty Acid/Retinol binding protein	*far-7*	K01A2.2	1.65	0.017
Q20489	Beta-lactamase-related domain-containing protein	*lact-1*	F46H5.8	1.592	0.002
Q95YE7	E3 ubiquitin-protein ligase PPP1R11 (Protein phosphatase 1 regulatory subunit 11)	C07H6.2	C07H6.2	1.581	0.023
O44572	Troponin I 4	*tni-4*	W03F8.1	1.571	0.015
Q17764	Phosphatidylcholine transfer protein (START domain-containing protein 2)	C06H2.2	C06H2.2	1.529	0.026
Q18286	Exocyst complex component 6 (Exocyst complex component Sec15)	*sec-15*	C28G1.3	1.513	0.027
Q8MXQ7	Threonylcarbamoyladenosine tRNA methylthiotransferase	Y92H12BL.1	Y92H12BL.1	1.503	0.035

B

E. coli + Lb. paragasseri (NN46, LG2055) (Day 5) < E. coli (Day 5)

Accession	Protein description	Gene name	Sequence name	Fold change	p-valure(raw)
Q18159	TIL domain-containing protein	C25E10.10	C25E10.10	0.426	0.007
Q9XUB2	Zinc finger protein	*mex-5*	W02A2.7	0.517	0.047
O16265	EGF-like domain-containing protein	F40A3.2	F40A3.2	0.591	0.039
Q20263	Probable Golgi transport protein 1 (Enlarged amphid sheath glia protein 1)	*eas-1*	F41C3.4	0.647	0.031
Q23359	CCCH-type zinc finger protein (Oocyte maturation defective protein 2)	*oma-2*	ZC513.6	0.659	0.025

Supplementary Figure 5 Proteome analysis of aged animals fed *E. coli* + *Lb. paragasseri*
Differentially expressed proteins between aged *E. coli*-fed and aged *E. coli* + *Lb. paragasseri*-fed animals. **(A)** Upregulated proteins. **(B)** Downregulated proteins.

Table S1 List of bacterial strains

Bacteria		Cultivation condition				
**Genus**	**Species**	**Broth**	**Time**	**Temperture**	**ID※**	**Nortes**
Escherichia (E)	*E. coli* (OP50)	Super broth	16 h	37 °C	-	
Bifidobacterium (B)	*B. adolescentis*	GAM broth (Nissui Pharmaceutical) supplemented with 1% glucose	18 h	37 °C	NN2	
	B. bifidum	"	16 h	"	NN5	
	B. breve	"	"	"	NN7	
	B. faecla	"	20 h	"	NN10	
	B. longum subsp. *longum*	"	16 h	"	NN15	
	B. longum subsp. infantis	"	19 h	"	NN16	
	B. pseudocatenulatum	"	18 h	"	NN20	
	B. pseudolongum subsp. *globosum*	"	"	"	NN23	
	B. thermophilum	"	16 h	"	NN25	
Lactobacillus (Lb)	*Lb. acidipiscis*	MRS broth (Becton Dickinson Co.)	20 h	37 °C	NN26	
	Lb. acidophilus	"	16 h	"	NN28	
	Lb. amylovorus	"	"	"	NN31	
	Lb. brevis	"	"	"	NN34	
	Lb. crispatus	"	"	"	NN35	
	Lb. delbrueckii subsp. *bulgaricus*	"	"	"	NN38	
	Lb. fermentum	"	"	"	NN42	
	Lb. helveticus	"	"	"	NN47	
		"	"	"	NN49	
	Lb. johnsonii	"	"	"	NN50	
	Lb. kefiri	"	20 h	"	NN53	
	Lb. mucosae	"	"	"	NN55	
	Lb. oris	"	16 h	"	NN58	
	Lb. parabuchneri	"	"	"	NN59	
	Lb. paracasei subsp. *tolerans*	"	"	"	NN60	
	Lb. paragasseri	"	"	"	NN45	
		"	"	"	NN46	Previously known as *L. gasseri* SBT2055
	Lb. plantarum	"	"	"	NN64	
	Lb. rhamnosus	"	"	"	NN68	
		"	"	"	NN111	
	Lb. sakei	"	20 h	"	NN71	
	Lb. salivarius subsp. *salicinius*	"	16 h	"	NN73	
	Lb. vaginalis	"	"	"	NN77	
Lactococcus (Lc)	*Lc. lactis* subsp. *lactis*	M17 broth (OXOID)	16 h	30 °C	NN79	
	Lc. raffinolactis	"	18 h	"	NN82	
Leuconostoc (Ls)	*Ls. citreum*	MRS broth (Becton Dickinson Co.)	18 h	30 °C	NN84	
	Ls. lactis	"	16 h	"	NN85	
	Ls. mesenteroides	"	24 h	25 °C	NN86	
			18 h	30 °C	NN87	
	Ls. mesenteroides subsp. *mesenteroides*	"	"	"	NN88	
	Ls. peudomesenteroides	"	"	"	NN90	
Limosilactobacillus(Lm)	*Lm. reuteri*	MRS broth (Becton Dickinson Co.)	16 h	37 °C	NN67	Previously known as *Lb. reuteri*
Pediococcus (P)	*P. acidilactici*	MRS broth (Becton Dickinson Co.)	16 h	30 °C	NN92	
	P. pentosaceus	"	"	37 °C	NN93	
	P. stilesii	"	"	30 °C	NN96	
Streptococcus (S)	*S. oralis*	MRS broth (Becton Dickinson Co.)	16 h	37 °C	NN97	
	S. parauberis	GAM broth (Nissui Pharmaceutical) supplemented with 1% glucose	23 h	30 °C	NN100	
	S. salivarius	MRS broth (Becton Dickinson Co.)	18 h	37 °C	NN101	
	S. thermophilus	M17 broth (OXOID)	16 h	"	NN103	
Weissella (W)	*W. cibaria*	MRS broth (Becton Dickinson Co.)	16 h	37 °C	NN105	
	W. confusa	"	"	"	NN108	
	W. paramesenteroides	"	"	30 °C	NN109	

※ID for this study only.
